# Accuracy Verification of Four-Dimensional CT Analysis of Knee Joint Movements: A Pilot Study Using a Knee Joint Model and Motion-Capture System

**DOI:** 10.7759/cureus.35616

**Published:** 2023-02-28

**Authors:** Takuya Adachi, Yuki Kato, Dai Kiyotomo, Katsushige Kawamukai, Shuzo Takazawa, Takahiro Suzuki, Youichi Machida

**Affiliations:** 1 Department of Sports Medicine, Kameda Medical Center, Chiba, JPN; 2 Department of Diagnostic Radiology, Tokyo Medical and Dental University, Tokyo, JPN; 3 Department of Radiology, Kameda Medical Center, Chiba, JPN; 4 Development of Design Division, Teijin Nakashima, Co. Ltd., Okayama, JPN

**Keywords:** ct based image analysis, four-dimensional computed tomography, joint kinematics, knee joints, motion analysis

## Abstract

Objective

This study aimed to use the optical motion-capture method to verify the accuracy of four-dimensional computed tomography (4D-CT) analysis of knee joint movement.

Methods

One static CT and three 4D-CT examinations of the knee joint model were obtained. The knee joint model was passively moved in the CT gantry during 4D-CT acquisitions. 4D-CT and static CT examinations were matched to perform 3D-3D registration. An optical-motion capture system recorded the position-posture of the knee joint model simultaneously with the 4D-CT acquisitions. Reference axes (X, Y, and Z directions) were defined based on static CT and applied to 4D-CT and the optical-motion capture system. Using the position-posture of the motion capture system as a reference standard, the position-posture measurements using 4D-CT were compared to these values, and the accuracy of the 4D-CT analysis of knee joint movements was quantitatively assessed.

Results

The position-posture measurements obtained from 4D-CT showed a similar tendency to those obtained from the motion-capture system. In the femorotibial joint, the difference in the spatial orientation between the two measurements was 0.7 mm in the X direction, 0.9 mm in the Y direction, and 2.8 mm in the Z direction. The difference in angle was 1.9° in the varus/valgus direction, 1.1° in the internal/external rotation, and 1.8° in extension/flexion. In the patellofemoral joint, the difference between the two measurements was 0.9 mm in the X direction, 1.3 mm in the Y direction, and 1.2 mm in the Z direction. The difference in angle was 0.9° for varus/valgus, 1.1° for internal/external rotation, and 1.3° for extension/flexion.

Conclusions

4D-CT with 3D-3D registration could record the position-posture of knee joint movements with an error of less than 3 mm and less than 2° when compared with the highly accurate optical-motion capture system. Knee joint movement analysis using 4D-CT with 3D-3D registration showed excellent accuracy for in vivo applications.

## Introduction

The prevalence of locomotor disorders is expected to increase with the aging of the global population. Elderly people with locomotor disorders may experience joint pain and require nursing care support, and locomotor disorders are reported to account for 12% of the "bedridden" cases. Knee osteoarthritis is a common locomotor disorder with an estimated prevalence of 16% of the world population, and its prevention and treatment are of substantial importance [1]. Surgical treatments such as total knee arthroplasty (TKA) have been performed for advanced knee osteoarthritis, and excellent results have been reported. However, some reports have also described TKA failure caused by infection, loosening, malalignment, patellofemoral issues, and instability [2-4].

Kinetically abnormal joint movement is involved in the progression of osteoarthritis and articular cartilage damage [5]. Therefore, accurate analysis of the six degrees of freedom of the knee joint is important for tracking the onset and progression of osteoarthritis and for assessing kinematic changes after medical interventions such as surgeries and rehabilitation. Several methods for evaluating dynamic knee joint movement have been reported to date, including the motion-capture method and the 2D-3D matching method. Motion-capture systems that measure "positions of human bodies, animals, and objects" and "time-series movements of joints" have recently gained acceptance. These systems are classified into the “optical type,” which is widely used, and the “non-optical type,” which includes mechanical motion-capture systems and magnetic systems. The principles of the optical motion-capture method were proposed by Rashid [6] in 1980. In this method, reflective markers are attached to an object, and its coordinates are measured using a camera. Because of its high accuracy in detection of the position and degree of free movement of objects, attempts have been made to use this method to evaluate gross joint movement. When the reflective markers are fixed to the object being evaluated, the accuracy of motion capture systems is sufficiently high. Still, the method for assessing joint motions by applying motion capture reflective markers directly to the skin may not necessarily show a high accuracy since there is a gap between the movements of bone and soft tissues (skin, subcutaneous fat, and muscle) [7-9]. Another method to improve accuracy loss due to soft tissue movement is to implant motion capture markers into the bone, but it is invasive and impractical for clinical application [10, 11].

The 2D-3D matching method is a technique for superimposing a projected image (2D data) created from 3D-CT (3D data) on an X-ray fluoroscopic image (2D data). This enables the measurement of distances and angles in three axial directions, and it has been used in computer-assisted navigation systems for the analysis of joint dynamics. This method enables highly accurate measurement of knee joint movement, especially for biplane analysis [10, 12-14]. However, evaluation of the patella with a few morphological features is difficult [11, 15], necessitating an additional CT scan to obtain three-dimensional morphological information [15].

Recent improvements in the multi-row structure of CT systems have enabled the acquisition of a wide range at high speed. Four-dimensional CT (4D-CT) adds a time axis to three-dimensional data and allows continuous CT imaging on a time-series basis. This method has widespread clinical application for assessments such as evaluation of blood flow dynamics [16-19]. In the field of orthopedic surgery, this method is expected to improve evaluations of joint dynamics and functions [20]. Unlike the 2D-3D matching method, 4D-CT can acquire three-dimensional morphological information as well as kinematic information. However, there are concerns about the accuracy of joint dynamics evaluations performed by 4D-CT due to the presence of motion artifacts and partial-volume effects [21]. In response to this concern, Oki et al. [22] reported a dynamic evaluation method using 3D-3D registration, which superimposes 3D-CT images created from static lower-limb full-length CT onto 4D-CT. However, no studies have been conducted to verify the position-posture evaluation accuracy of knee joint movement using 4D-CT.

We hypothesized that 4D-CT using 3D-3D registration would enable highly accurate analysis of knee joint movement. Thus, the purpose of this study was to verify whether high-precision analysis of knee joint movement is possible with 4D-CT using 3D-3D registration. We fixed optical-motion capture reflective markers on the bone model and manually performed knee joint motion. We simultaneously recorded the knee motion using 4D-CT and the high-precision optical-motion capture described above. We evaluated the accuracy of the knee joint motion measured by 4D-CT using the motion capture as a reference standard.

If 4D-CT enables a highly accurate assessment of knee joint dynamics, it may be possible to objectively detect abnormal joint motion in osteoarthritis patients at an early stage. Early diagnosis would allow for early intervention in knee osteoarthritis. Application to knee joint dynamics before and after knee surgery is also considered. By comparing the knee joint dynamics objectively captured by 4D-CT before and after surgery, it is expected to lead to early detection of poor prognosis patients and improvement of surgical procedures and rehabilitation to recover more appropriate knee joint motion.

This article was previously posted to the Research Square preprint server on September 27, 2021.

## Materials and methods

**Figure 1:**
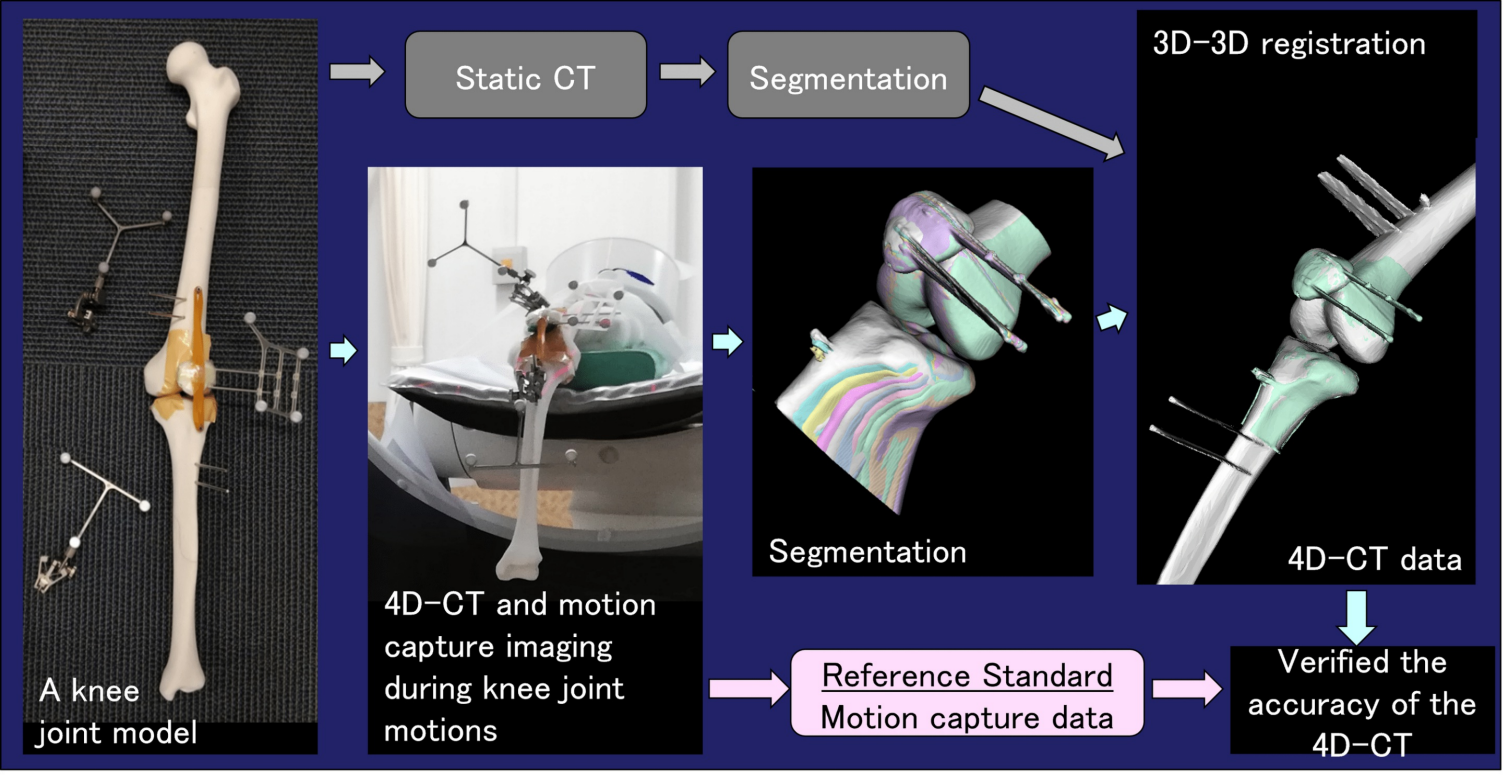
Process flow from CT acquisition to dynamic analysis Static of the entire knee joint model and 4D-CT of the knee joint motion were acquired, and 3D-3D registrations were performed between them to measure the position and posture of the knee joint motion from the 4D-CT. The position and posture of the knee joint motion were measured simultaneously using the optical-motion capture system during the 4D-CT scans were performed, and the results were used as a reference standard. The accuracy of the 4D-CT was verified by comparing the positional orientation measured by the 4D-CT with the reference standard.

A knee-joint model was used as the subject. The model was passively moved inside the gantry of the CT device, and 4D-CT data were obtained, which were matched with the static CT data of the entire knee joint model (from the femoral head to distal tibial epiphysis) to perform 3D-3D registration. An optical motion-capture system was used to verify the accuracy of 4D-CT data with 3D-3D registration (Figure 1).

Bone model creation

**Figure 2:**
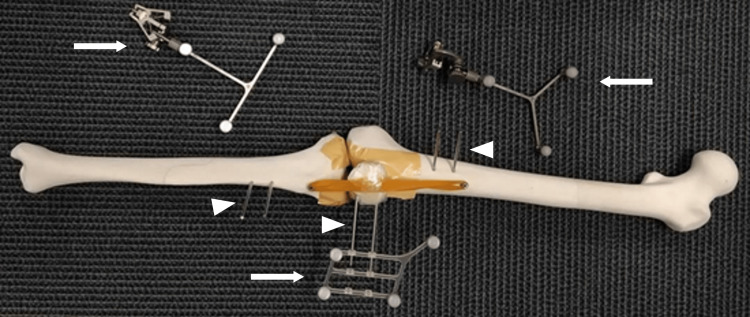
Human knee bone model The bone models were connected with rubber bands, and two tracker pins (arrowheads) were attached to each bone model for the optical-motion capture system. The metal fixtures that hold the reflective markers (arrows) were connected to the tracker pins, and the optical-motion capture system was applied to capture knee joint motions.

A human knee skeleton model, including the femur, tibia, and patella, was manufactured by the laminate-shaping method using ZPrinter 150 (3D Systems, Inc., Rock Hill, SC, USA), yielding the same shape as “SAWBONES SKU: 1148-3” (Pacific Research Laboratories, Inc., Sawbones, WA, USA). To reproduce the knee joint fissures in the bone model, cushioning materials with a thickness of approximately 5 mm were taped to the articular surface of the tibial model and the patellofemoral joint of the femur model to mimic the articular cartilage. Rubber bands were attached to both ends (distal and proximal) of the patella model, with the distal rubber band fixed to the anterior surface of the femoral model and the proximal rubber band to the anterior surface of the tibial model. Using this approach, the three bone models that constituted the knee joint were connected. Tracker pins with a diameter of 3 mm were placed in each bone model to attach the tracker used in the optical-motion capture system (Figure 2).

CT acquisition

**Figure 3:**
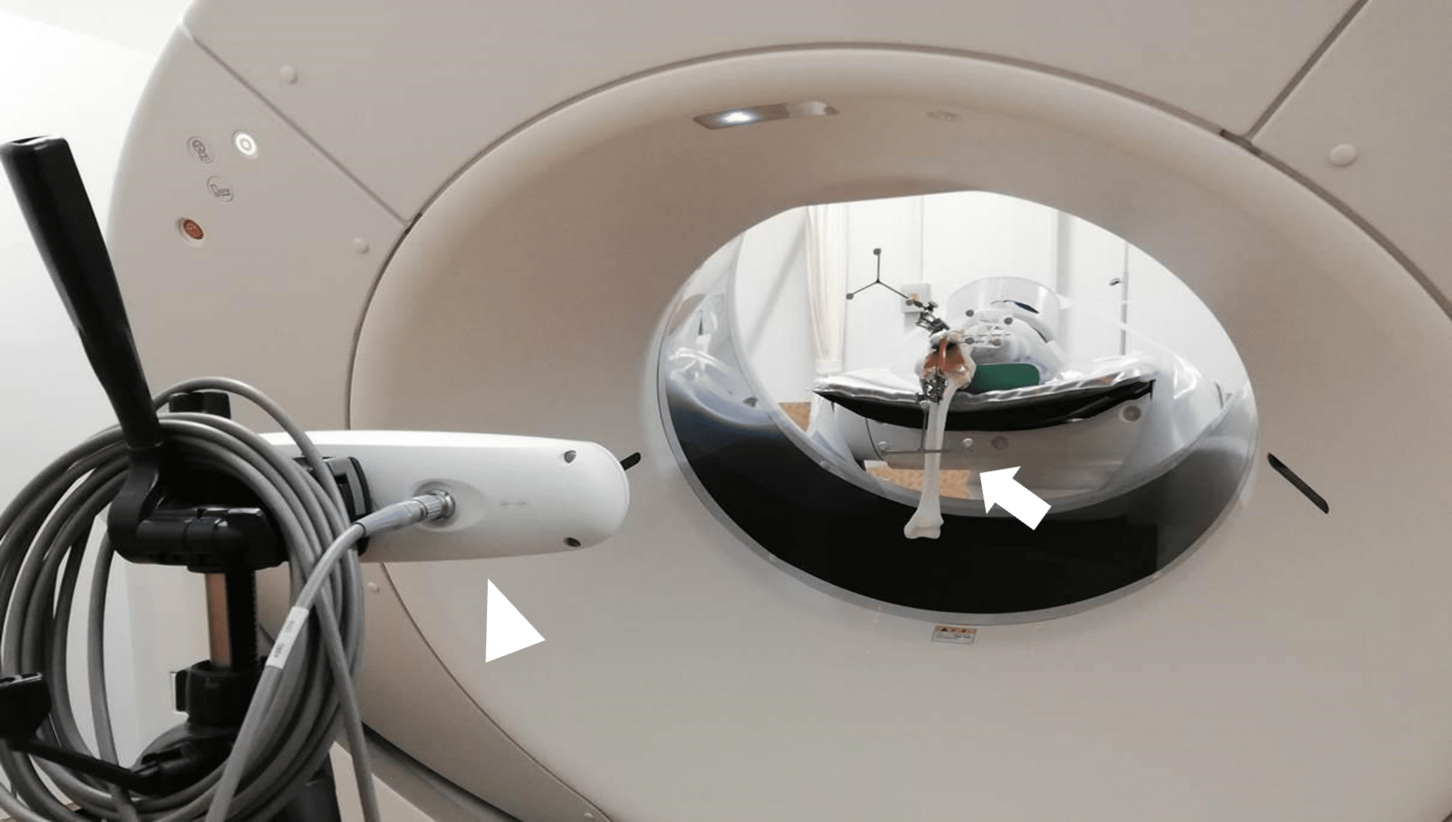
Knee joint model in the CT gantry and the optical-motion capture system The knee joint model was fixed on the CT gantry (arrow), and the optical-motion capture system was placed facing the knee joint model (arrowhead).

A 320-slice multidetector CT (Aquilion One, Canon Medical Systems, Otawara, Japan) was used for CT imaging. Table 1 shows the imaging parameters for static CT images and 4D-CT images. First, a static CT image of the entire knee joint model was taken in the extension position (0 degrees). Next, the femoral side was fixed to the table such that the center of knee joint movement was located at the center of the CT imaging range. With the CT gantry tilted at 30°, 4D-CT scans were obtained with a rotation time of 10.5 s while the knee joint model was passively flexed from 0° to 90° by an experimenter. Passive knee joint movements were performed in approximately 10.5 s, with the obtained 4D-CT data consisting of 20 volume scans. During 4D-CT scanning, knee joint motion started in the extended position (0 degrees) and performed a series of knee flexion, extension, and another flexion. The positional posture of the knee joint model was simultaneously recorded by the optical motion capture system at the same time as the 4D-CT imaging (Figure 3). The entire process of CT imaging as described above was repeated three times.

**Table 1:** Scanning parameters for static CT and 4D-CT data acquisitions

	Gantry tilt (degrees)	Section thickness (mm)	Range (mm)	Tube potential (kV)	Effective mAs (mAs)	Rotation time (s)	Acquisition interval	CT Dose Index Volume (mGy)	Dose-length product (mGy-cm)
Static	0	1.0×40	1000	120	9	0.5	NA	0.7	49
4D-CT	30	0.5×160	160	80	87	0.5	Continuous	31.0	495.6

Surface reconstruction and reference axis

**Figure 4:**
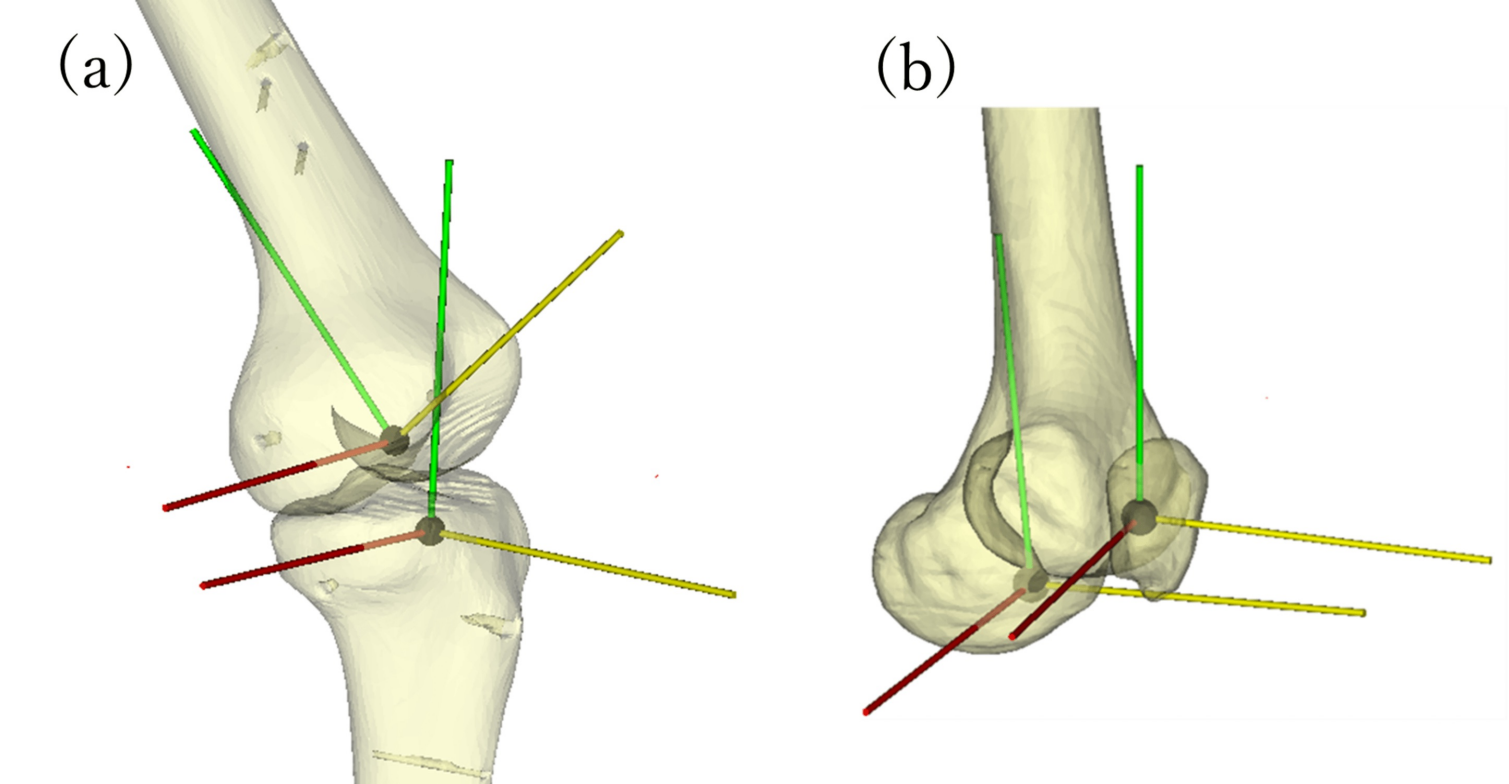
The reference axis of the knee joint model on the static CT (a) Local coordinate systems of the femur and tibia, (b) local coordinate systems of the femur and patella.

The 4D-CT images in DICOM format were imported into a 3D model construction software (N-View, Teijin-Nakashima Medical Co., Ltd., Okayama, Japan), and 3D models of the femur, tibia, and patella, which will be referred to as the “4D-CT model” hereafter, were constructed for each of the 20 volume scan acquisitions. Similarly, 3D models of the femur, tibia, and patella, which will be referred to as the “static CT model” hereafter, were constructed from the static CT images along the entire length of the lower limbs of the knee joint model. Simultaneously, a reference axis was defined on the static CT model (Figure 4). In the 4D-CT model, the coordinate system was set for each volume scan based on the reference axis.

In the coordinate system of the femur, the origin was defined as the point where the center of the femoral medullary canal intersects with the distal femur. This is generally the point at which the intramedullary rod is inserted during total knee arthroplasty. The Z-axis was defined as a line running through the femoral head center (hip center) and the origin. The X-axis was defined as a line parallel to the projected line of the surgical epicondylar axis (SEA), which is a line that connects the lateral femoral epicondyle and the sulcus of the medial femoral epicondyle in the femoral transverse plane perpendicular to the Z-axis. The Y-axis was defined as the line perpendicular to the X and Z-axes. In this coordinate system, the medial direction on the X-axis, the anterior direction on the Y-axis, and the proximal direction on the Z-axis were defined as positive.

In the coordinate system of the tibia, the origin was defined as the point 10 mm distal to the lateral tibial articular surface on the center of the tibial medullary canal. This is generally the central insertion point for tibial implants in total knee arthroplasty. The Z-axis was defined as the line connecting the origin and the center of the talocrural joint. The Y-axis was defined as the line passing through the origin, parallel to the line which connects the medial border of the patellar tendon attachment of the tibial tuberosity and the posterior cruciate ligament tibial attachment. The X-axis was defined as the line perpendicular to the Y and Z axes. In this coordinate system, the medial direction on the X-axis, the anterior direction on the Y-axis, and the proximal direction on the Z-axis were defined as positive.

In the coordinate system of the patella, the origin was defined as the midpoint of the long (mediolateral) and short (proximal-distal) axes of the coronal plane 8 mm anterior and parallel to the articular surface of the patella. The Z-axis was defined as a line passing through the origin of the patella, parallel to the line connecting the patella’s base and the patella’s apex. The X-axis was defined as a line through the origin, parallel to the femoral X-axis. The Y-axis was defined as the line perpendicular to the X and Y-axes. In this coordinate system, the medial direction on the X-axis, the anterior direction on the Y-axis, and the proximal direction on the Z-axis were defined as positive.

3D-3D registration

The imaging range of 4D-CT can be as small as 16 cm wide in the craniocaudal direction, and the field of view (FOV) was 512 x 512 for both static CT and 4D-CT. The center of the femoral head and the center of the ankle joint, which are the reference points on the functional axis, are outside the imaging range of 4D-CT. In addition, the reference axis for evaluating the position-posture needs to be re-established for each volume scan. Position-posture evaluation with the 4D-CT model alone can be inaccurate for these two reasons. Therefore, in this study, the reference axis defined in the static CT model was applied to the 4D-CT model by surface-matching the two models in order to calculate the accurate position-posture of the 4D-CT model. Surface matching was completed using a software for editing polygon meshes (GOM Inspect 2018, GOM GmbH, Braunschweig, Germany).

Precision validation

For each volume scan, the position-posture expressed in the femur model coordinate system was calculated with reference to the tibia model coordinate system as the “FT position-posture.” Similarly, for each volume scan, the position-posture expressed in the femur coordinate system based on the coordinate system of the patella model was also calculated as the “PF position-posture.” The position of each volume scan was determined by determining the positional relationship between the origins of the coordinate system between volume scans, and the posture was obtained by angle-resolving the "distortion" between the coordinate systems.

The movement angle of the femur relative to the tibia is described in terms of varus/valgus, internal/external rotation, and extension/flexion. Varus/valgus indicates the angle of the femoral X-axis projected onto the XZ plane of the tibial coordinate system (Angle between femur X-axis and tibia X-axis). Internal/external rotation indicates the angle of the femoral X-axis projected onto the XY plane of the tibial coordinate system (Angle between femur X-axis and tibia X-axis). Extension/flexion indicates the angle of the femoral Y-axis projected onto the YZ plane of the tibial coordinate system (Angle between femur Y-axis and tibia Y-axis).

The movement angle of the femur relative to the patella is described in terms of varus/valgus, internal/external rotation, and extension/flexion. Varus/valgus indicates the angle of the femoral X-axis projected onto the XZ plane of the patella coordinate system (Angle between femur X-axis and tibia X-axis). Internal/external rotation indicates the angle of the femoral X-axis projected onto the XY plane of the patellar coordinate system (Angle between femur X-axis and tibia X-axis). Extension/flexion indicates the angle of the Y-axis of the femur projected onto the YZ plane of the patellar coordinate system (Angle between femur Y-axis and tibia Y-axis).

The position-posture measurement of the bone model by the optical-motion capture system (N-navi, Teijin Nakashima Co., Ltd.) was performed in synchronization with 4D-CT imaging. For measurements with the optical-motion capture system, the same CT model and alignment definition as those adapted for 3D-3D registration were used. This measurement of the position and orientation of the simulated bone by the optical-motion capture system facilitated comparison of the position and orientation calculated by 3D-3D registration with the same criteria. Since the optical-motion capture system used in this study had an error of 0.6 mm and within 1 degree according to a previous study [23], the position and orientation measurement by this optical-motion capture system was used as the reference standard. In each 4D-CT volume scan, the difference in position and posture (coordinates, varus/valgus, internal rotation/external rotation, extension/flexion) from the optical-motion capture system was calculated. The same analysis was performed on each of the three acquisitions of CT data. The accuracy of position-posture evaluation by 4D-CT was verified by calculating the average of absolute value differences.

## Results

**Figure 5:**
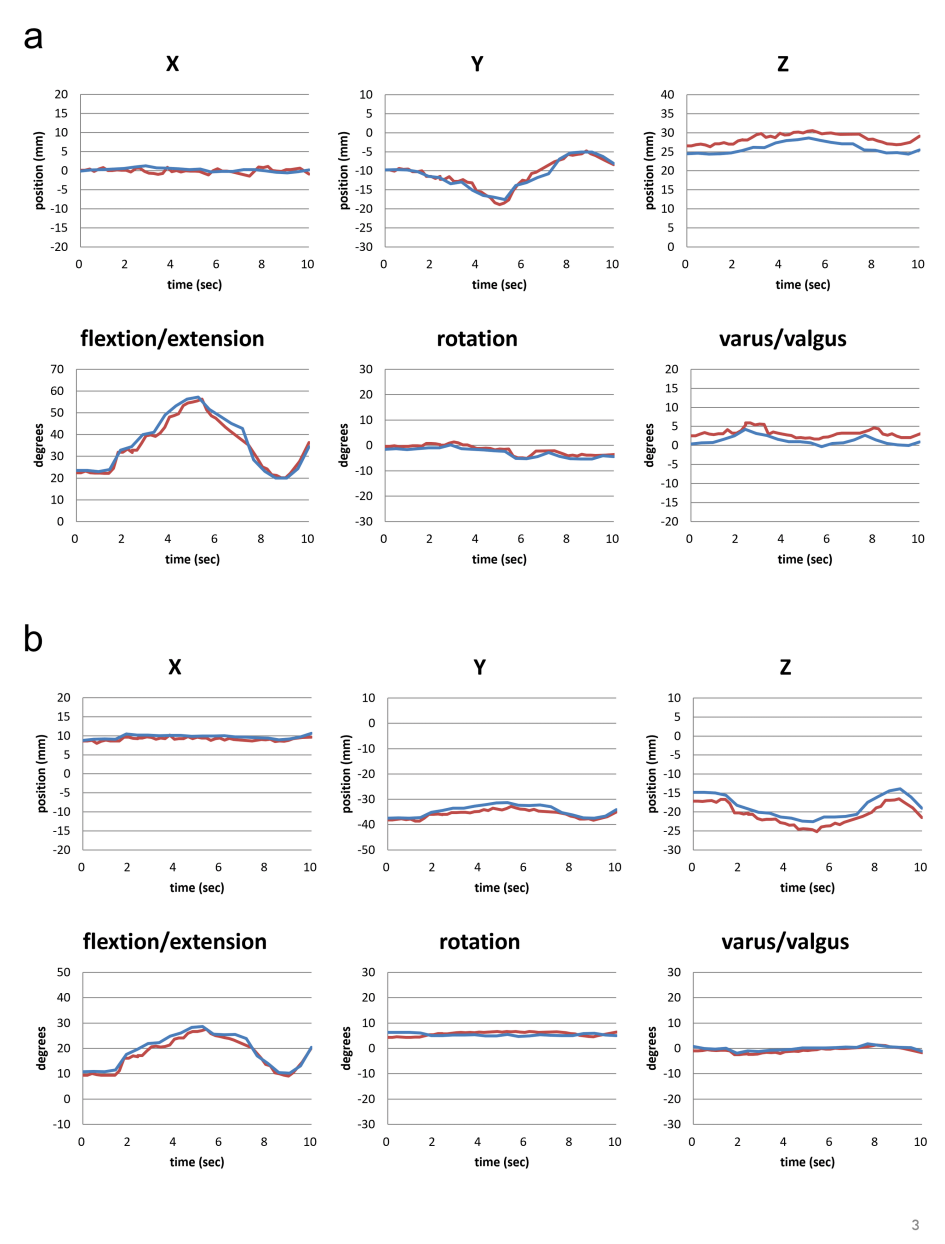
Changes in the position-posture measured by 4D-CT (blue line) and the optical-motion capture system (red line) in the FT joint (a) and the PF joint (b) The 4D-CT and optical motion capture systems showed similar movement patterns for FT and PF joints in both positions (X, Y, Z coordinates) and posture (flexion/extension, rotation, varus/valgus).

**Figure 6:**
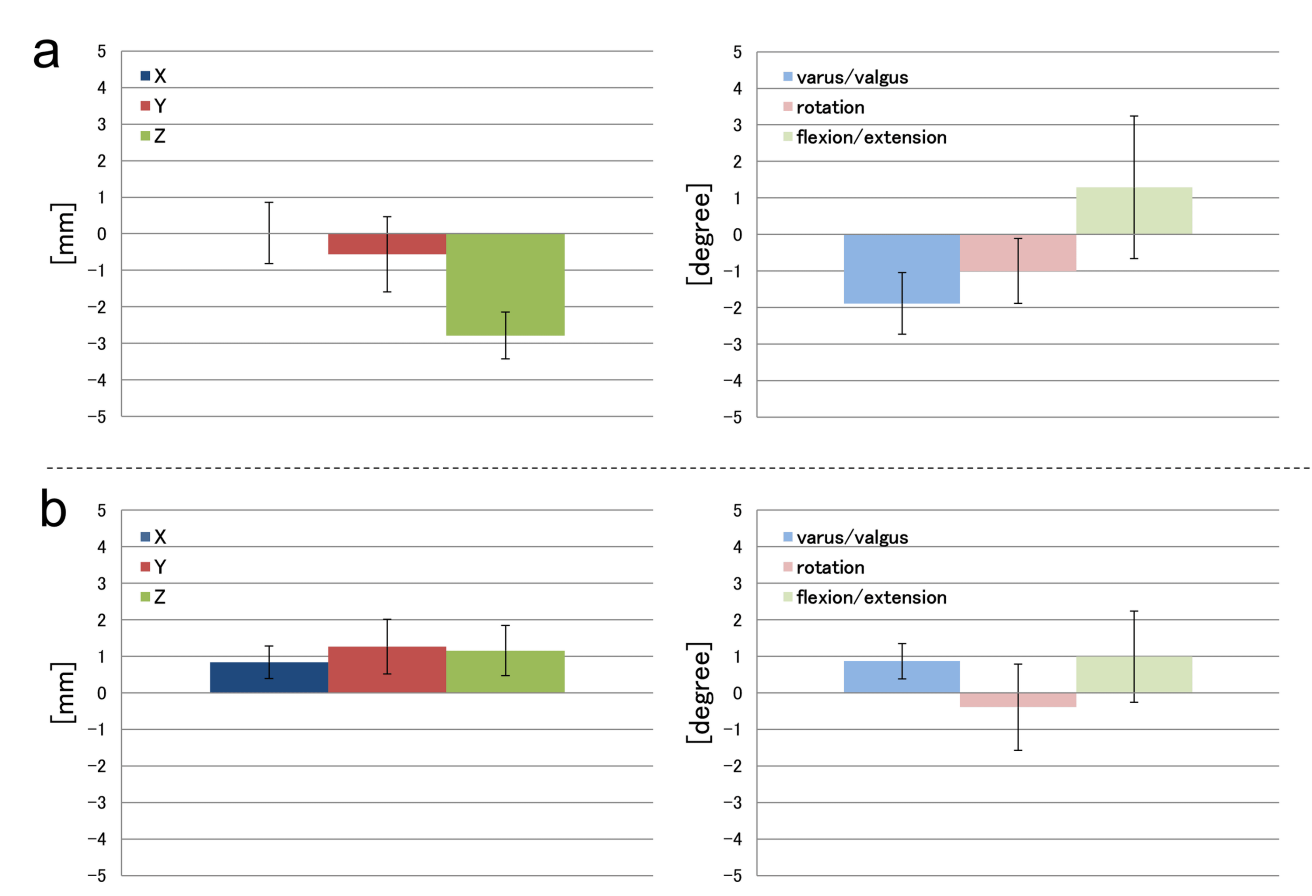
Differences between 4D-CT and the optical-motion capture system in the FT joint (a) and the PF joint (b) Differences are shown as relative values, indicating errors of up to 2.8 mm and 1.8° for the FT joint, and up to 1.3 mm and 1.8° for the PF joint. Thus, 4D-CT could measure the position and orientation with high accuracy.

Figure 5 shows one of the three temporal position-posture measurements of 4D-CT and the optical-motion capture system for the tibiofemoral joint (FT joint) (Figure 5a) and patellofemoral joint (PF joint) (Figure 5b). Figure 6 shows the difference in position-posture measurements between 4D-CT and the optical-motion capture system for the FT joint (Figure 6a) and PF joint (Figure 6b).

The position-posture obtained from 4D-CT showed similar tendency as that obtained from the optical-motion capture system. In the FT joint, the difference in spatial orientation between the two measurements was 0.7 mm in the X direction, 0.9 mm in the Y direction, and 2.8 mm in the Z direction. The difference in angle was 1.9° in the varus/valgus direction, 1.1° in the internal/external rotation, and 1.8° in the extension/flexion (Figure 6a). In the PF joint, the difference between the two measurements was 0.9 mm in the X direction, 1.3 mm in the Y direction, and 1.2 mm in the Z direction. The difference in angle was 0.9° for varus/valgus, 1.1° for internal/external rotation, and 1.3° for extension/flexion (Figure 6b).

**Figure 7:**
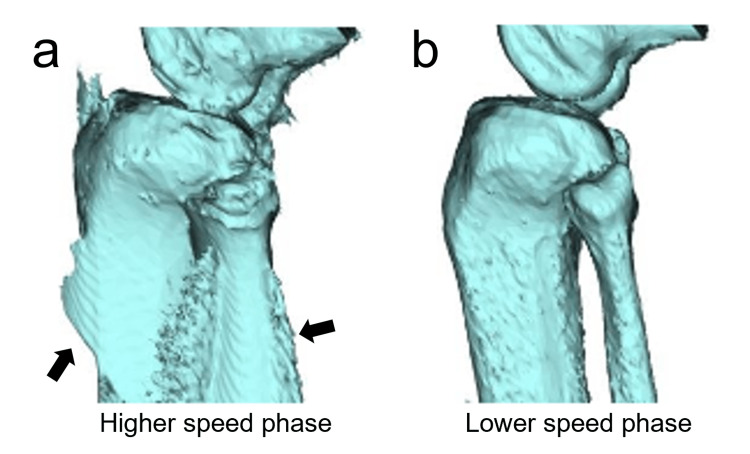
3D-CT model reconstructed from 4D-CT data Some motion artifacts (arrows) occurred in the tibia and fibula during the mid-term flexion and extension movements (a), where the movement speed was high. In contrast, when the movement speed was relatively low (that is, when the movement per unit time was small), no artifact was observed (b).

Since the subject itself moved while the gantry rotated, the 4D-CT data exhibited a partial-volume effect and motion artifacts (Figure 7). Therefore, some artifacts were included in the bone model constructed by the binarization method from this 4D-CT image. In particular, artifacts were most noticeable in the middle of the flexion and extension movements, which had a high movement speed, and the afterimage of movements was confirmed in the bone axis of the tibia, which showed the largest movement (Figure 7).

## Discussion

In the present study, knee joint movement of the bone model was analyzed by matching 4D-CT and static CT scans of the entire length of the lower limbs by using 3D-3D registration. In order to examine the analysis accuracy, this method was compared with the high-precision optical-motion capture system. The findings revealed that this was a highly accurate method for evaluating knee joint movements, with an error of less than 3 mm and less than 2°.

To our knowledge, reports on the accuracy of 4D-CT have been primarily focused on radiotherapy [24-26]. Lee et al. validated the accuracy of 4D cone-beam CT for radiotherapy by using a phantom and reported that the error was less than 2 mm when the respiratory movement was up to 30 mm [21]. The present study is the first to analyze the accuracy of 4D-CT in the joint area, and it showed that this technique showed the same accuracy as it did in the evaluation of respiratory movement.

Several reports in the field of radiotherapy have referred to artifacts in 4D-CT, demonstrating that artifacts affect lung function imaging and tumor volume assessment [24-26]. A similar problem may affect 4D-CT of the bone joint area for evaluating joint movement. In this study, highly accurate evaluation was possible by performing 3D-3D matching. However, it is unclear whether joint motion was evaluated correctly in the previous reports that did not perform registration with static CT.

In assessments using 4D-CT, the imaging range is limited to the area around the knee joint, making the alignment of the entire lower limbs unclear. In addition, the reference axis for evaluating the position and orientation must be provided for each volume scan, which may introduce errors. To avoid these problems, we measured knee joint movement using 3D-3D registration, matching the static CT model of the full length of the lower limbs with 4D-CT in accordance with the method described by Oki et al. [22]. Conventional methods such as 2D-3D matching have been considered to be particularly difficult to analyze the patella in the dynamics of the knee joint [12]. However, 4D-CT enabled the position and posture of the patella to be determined from three-dimensional data, allowing measurements of the patellofemoral joint with the same high accuracy as the femoral tibial joint.

Various methods have been used for the analysis of knee joint dynamics, and the accuracy of these methods has also been reported. However, while this study investigated the accuracy of analyzing passive ROM movement in the non-weight-bearing position, previous reports performed dynamic analysis during walking and running. Therefore, comparisons between the present and previous studies are difficult. In dynamic analysis using body surface markers, the deviation from the bone movement due to the movement of the skin is a major concern. Sati et al. [27] reported a 2-17 mm gap between skin and bone in medial and lateral femoral condyles. Therefore, high accuracy cannot be expected using this method. Another common method is to attach external markers directly to the bone using percutaneous pins. However, Holden et al. [28] reported an error of up to 10 mm and 8° using this method. Accuracy analyses using fluoroscopy have also been performed. Hoff et al. [13] achieved a very high accuracy of 2.25 mm and less than 0.035°. However, this analysis method can only be applied to the knee in which the implant is inserted. The analysis of knee joint movement by 4D-CT in this study can be applied to patients who have not undergone knee joint surgery to receive implants. 4D-CT can be expected to yield high accuracy in evaluation of actual patients.

The matching error between the 4D-CT and CT models can be explained by the fact that the 4D-CT model includes the effect of movement, and that the optical-motion capture system used for reference also contains an error. The result that measurement error in 4D-CT was relatively large in the middle portion of the knee extension/flexion was caused by the faster movement of the bone model. Thus, the accuracy of analysis by 4D-CT can be improved by reducing the motion speed of knee joint movement.

This study had several limitations. First, this study involved experimental verification using a bone model, which evaluated the dynamics of passive motion in the non-weight-bearing position. Verification with actual clinical patients can be expected in future studies. However, conventional CT equipment only allows participants to be evaluated in the non-weight-bearing position, which is a major disadvantage in any study. Upright CT, which allows a patient to stand, has been recently developed at the research level. While evaluations in the weight-bearing position are expected to be facilitated by this new CT technique, clinical application of the new technology will take more time [29]. Therefore, it is meaningful to pursue a highly accurate joint dynamics evaluation method in a widely used device. Second, the optical-motion capture system contained inherent errors, which may have affected the analysis accuracy of this study. This optical-motion capture system, which is also used in surgery, has been used in many studies analyzing knee joint motion [30] and was shown to be extremely accurate, with an error of 0.6° and within 1 mm, indicating only a minimal effect on the scale of knee joint movement [23]. However, there may be problems when using this technique for small joints, because small errors can have a relatively large effect in small joints when compared to large joints. Third, because this pilot study evaluated the accuracy of 4D-CT using only three imaging sessions with a bone model, we could not add statistical analysis to the study. However, we believe that the accuracy of the results of this study is high, given that the same bone model was used for all three imaging sessions, and there was no variation in the imaging subjects. Finally, both 4D-CT and general CT examinations are associated with the risk of radiation exposure. To avoid this risk, patients’ gonads should be protected, and possible measures should be taken to reduce radiation exposure when the patients undergo CT examinations. Further studies should be made using an appropriate 4D-CT imaging protocol.

## Conclusions

In this study, the accuracy of knee joint analysis using 4D-CT with 3D-3D registration was determined using the optical motion-capture system. The knee joint movement analysis using 4D-CT with 3D-3D registration showed excellent accuracy for in vivo applications.

In the future, comparing preoperative and postoperative 4D-CT findings in patients with osteoarthritis and knee ligament injuries could provide an assessment of whether surgery has resulted in the acquisition of normal knee joint motion. This dynamic imaging evaluation may contribute to the early detection of poor prognosis after surgery and early diagnosis of knee osteoarthritis.
