# Correction: Accuracy Verification of Four-Dimensional CT Analysis of Knee Joint Movements: A Pilot Study Using a Knee Joint Model and Motion-Capture System

**DOI:** 10.7759/cureus.c294

**Published:** 2025-09-16

**Authors:** Takuya Adachi, Yuki Kato, Dai Kiyotomo, Katsushige Kawamukai, Shuzo Takazawa, Takahiro Suzuki, Youichi Machida

**Affiliations:** 1 Department of Sports Medicine, Kameda Medical Center, Chiba, JPN; 2 Department of Diagnostic Radiology, Tokyo Medical and Dental University, Tokyo, JPN; 3 Department of Radiology, Kameda Medical Center, Chiba, JPN; 4 Development of Design Division, Teijin Nakashima, Co. Ltd., Okayama, JPN

The Ethics Statement and Conflict of Interest Disclosures section has been updated to disclose the following:

**Financial relationships:** Dai Kiyotomo declare(s) employment from Teijin Nakashima Co., Ltd. This study was conducted as a collaborative research project with Teijin Nakashima Co., Ltd. The company’s research-use, non-commercial navigation software (N-view) was utilized. Dr. Kiyotomo prepared the knee joint model and performed the 3D-CT model reconstruction; data analysis was conducted by Drs. Yuki Kato (corresponding author) and Adachi. Teijin Nakashima Co., Ltd. had no role in the study design, decision to publish, or manuscript preparation.

